# The relationship between serum vitamin D levels and sleep quality in fixed day indoor field workers in the electronics manufacturing industry in Korea

**DOI:** 10.1186/s40557-017-0187-7

**Published:** 2017-06-24

**Authors:** Young Saeng Jung, Chang Ho Chae, Young Ouk Kim, Jun Seok Son, Chan Woo Kim, Hyoung Ouk Park, Jun Ho Lee, Young Hoo Shin, Ho Sung Kwak

**Affiliations:** Department of Occupational and Environmental Medicine, Samsung Changwon Medical Center, Sungkyunkwan University School of Medicine, Changwon City, Republic of Korea

**Keywords:** Fixed day workers, Serum vitamin D, Sleep quality

## Abstract

**Background:**

Although recent studies have investigated the influence of vitamin D on sleep patterns, there is a lack of research on the relationship between vitamin D and sleep patterns in Korean workers. This study focused on the relationship between serum vitamin D levels and sleep in fixed day indoor field workers in the electronics manufacturing industry in Korea.

**Methods:**

The 1472 subjects who were included in this study were selected from fixed day workers in the electronics manufacturing industry who had received a worker’s special health examination at a hospital in Changwon, South Gyeongsang Province between January 2015 and December 2015. Nighttime workers and those who showed symptoms of depression were excluded from this study. The sociodemographic and lifestyle variables of the participants were investigated, including age, sex, marital status, level of education, body mass index, smoking habits, alcohol consumption habits, and regular exercise. Work-related factors were evaluated, such as employee tenure and occupational stress. Serum 25-hydroxyvitamin D was measured as an indicator of vitamin D levels, and quality of sleep was evaluated using the Pittsburgh Sleep Quality Index (PSQI) translated into Korean.

**Results:**

The subjects had a mean serum vitamin D level of 13.70 ± 5.93 ng/mL. Vitamin D deficiency, defined as a serum vitamin D level of <10 ng/mL, was found in 24.8% of males and significantly more frequently in females (47.6%). Poor sleep quality was reported by 19.8% of participants with serum vitamin D levels ≥10 ng/mL and by 21.7% of those with serum vitamin D levels <10 ng/mL, which was a significant difference (*P* = .007). Multiple logistic regression analysis adjusting for significant variables found that poor sleep quality was more likely in those with vitamin D deficiency than those with higher serum vitamin D levels (odds ratio = 1.36; 95% CI, 1.01–1.82). A comparison of serum vitamin D levels and PSQI components showed that the mean scores for subjective sleep quality, sleep latency, and sleep duration were significantly higher in the vitamin D-deficient participants, indicating that the vitamin D-deficient participants had poorer sleep quality.

**Conclusions:**

This study investigated serum vitamin D levels in fixed day indoor field workers in the manufacturing industry in Korea and analyzed the relationship of vitamin D deficiency with sleep quality. A significant correlation was found between serum vitamin D deficiency and poor sleep quality. Based on the results of this study, sleep disorder management for workers can be improved by providing regular examinations checking their serum vitamin D levels and supplying vitamin D to workers with serum vitamin D deficiency to enhance their quality of sleep.

## Background

Vitamin D deficiency has become a worldwide problem, is increasing in prevalence [1, 2], and is found in Korea as well. A study on serum vitamin D deficiency in Koreans [3] that analyzed data provided by the Korean National Health and Nutrition Examination Survey (KNHANES 2010–2011) reported that the mean serum vitamin D level in Koreans was 17.38 ng/mL, which is lower than has been reported in other countries: the population mean in the United States was found to be 22.08 ng/mL based on the National Health and Nutrition Examination Survey data [4]; the mean in Canada was 27.08 ng/mL, according to the Canadian Health Measures Survey [5]; and the mean in Japan was 22.80 ng/mL, based on a study conducted of serum vitamin D levels among the general population [6]. Vitamin D deficiency can occur due to spending a large proportion of living or working hours indoors [2] and as a result of sunscreen use [7]; additionally, serum vitamin D levels are known to vary according to age [8], race/ethnicity and skin color [9], season, altitude, and latitude of the sun [10].

The synthesis of vitamin D mostly takes place on the epidermis of the skin when exposed to ultraviolet radiation [11]. Vitamin D mainly functions in the intestinal absorption of calcium and phosphorus [12], and is essential in maintaining healthy skeletal development. Without enough vitamin D, the risk of osteoporosis and bone fractures is elevated, and vitamin D deficiency may cause rickets in children or osteomalacia in adults [13]. Not only is vitamin D widely known for its role in maintaining skeletal health, but studies have also reported it to play a role in cardiovascular disease, high blood pressure [14], diabetes [15], and obesity [16]. Furthermore, studies have revealed connections between abnormal vitamin D levels and other non-skeletal diseases such as abnormal cell proliferation and differentiation, tumors [17], and immune system modulation [18]; thus, vitamin D is very significant from a clinical and population health perspective.

Recent studies have focused on the less commonly known roles of vitamin D, such as its influence on sleep. The mechanism of vitamin D and its effect on sleep is likely to play a major role in the brainstem, which controls sleep. This hypothesis is supported by evidence of vitamin D receptors in parts of the brainstem [19]. Sleep disorders have rapidly increased in some developed countries [20], and the number of sleep disorder cases in Korea is also increasing. According to an analysis of the recent 5-year data provided by the Korea National Health Insurance Service on medical expenses spent on sleep disorders, a 57.5% increase took place in the most recent 5 years, from 280,000 persons in 2010 to 450,000 persons in 2015, which confirms that sleep disorders have become a serious health problem for Koreans [21].

Previous studies on the correlations of serum vitamin D levels and sleep duration have been conducted in Korea using KNHANES data collected between 2010 and 2011: one focused on individuals 19 years of age and older in the general population [22], and the other concentrated on the elderly population 65 years of age and older [23]. Although these 2 studies showed a difference in degree, they both came to the conclusion that lower serum vitamin D levels were associated with a significantly higher risk of short sleep duration.

To the knowledge of the authors, no research on possible correlations between vitamin D levels and sleep has yet been conducted on Korean workers. Their work environment, which involves indoor work during the day, could give them less exposure to sunlight, thus making them more susceptible to serum vitamin D deficiency than the general population and potentially more likely to experience sleep disorders caused by work-related factors such as occupational stress. It was hypothesized that research on vitamin D levels and sleep disorders in workers would show different results from those reported in previous studies conducted on the general population. This study aimed to investigate the relationship between serum vitamin D levels and sleep in fixed day indoor field workers in the electronics manufacturing industry in Korea.

## Methods

### Subjects

A total of 6316 indoor field workers in the electronics manufacturing industry who had received a worker’s special health examination at a university hospital in Changwon, South Gyeongsang Province between January 2015 and December 2015 participated in this study. Ultimately, 1472 participants were selected to be included in the study after excluding those affected by exogenous variables (It is possible to reduce the internal validity of the study as a confounding variable that affects dependent variables.), such as nighttime workers (*n* = 4578) and those who showed symptoms of depression (16 points or higher on the Center for Epidemiologic Studies Depression Scale; *n* = 201), as well as 65 participants who had missing values in their examination results or data.

### General characteristics

Sociodemographic and lifestyle variables of the participants were investigated, including age, sex, marital status, level of education, body mass index, smoking habits, alcohol consumption habits, and regular exercise habits. Work-related factors, such as employee tenure and occupational stress, were also evaluated. The participants ranged in age from 19 to 39 years and were grouped by age as younger than 30 or 30 years and older. Their marital status was classified as unmarried or married. Participants were classified into 2 groups according to educational attainment: high school graduation or less and bachelor’s degree or higher. They were classified according to body mass index as <25.0 kg/m^2^ or ≥25.0 kg/m^2^, following the World Health Organization classification based on Asia-Pacific population standards [24]. Participants were classified by smoking habits as current smokers or nonsmokers (including never smokers and former smokers). Alcohol consumption habits were classified using the KNHANES high-risk drinking classifications [25], with the high-risk drinking group classified as those who drank more than 2 times a week with an average of 7 drinks consumed in any 1 drinking session (5 drinks for females). Regular exercise habits were classified according to exercise intensity was disregarded, and exercise frequency was classified as ≥3 times a week and <3 times a week. Employee tenure was divided into <5 years, 5–9 years, and ≥10 years. Occupational stress was measured using the Korean Occupational Stress Scale-Short Form (KOSS-SF) [26]. The KOSS-SF is composed of 7 sections (job demand, insufficient job control, interpersonal conflict, job insecurity, organizational system, lack of reward, occupational climate) with 24 questions. Using the KOSS-SF median reference values, occupational stress was categorized into high occupational stress and low occupational stress. The research was conducted seasonally to account for seasonal changes in serum vitamin D levels; spring was defined as March–May, summer as June–August, autumn as September–November, and winter as December–February.

### Measurement of serum vitamin D levels

This study measured serum 25-hydroxyvitamin D, which is the best indicator of vitamin D conditions in the body [27]. Specimens were kept frozen until they were analyzed using the electrochemiluminescence immunoassay method on a Modular E apparatus (Hitachi Co, Tokyo, Japan). Like previous studies [28–31], this study used 10 ng/mL as the cut-point for serum vitamin D deficiency, and classified subjects into groups of those with levels <10 ng/mL and ≥10 ng/mL.

### Sleep quality

The participants’ quality of sleep was evaluated using the Pittsburgh Sleep Quality Index (PSQI) translated into Korean [32]. This tool evaluates sleep quality during a 1-month period and contains 7 components: subjective sleep quality, sleep latency, sleep duration, sleep efficiency, sleep disturbance, use of sleeping medication, and daytime dysfunction. The scores for each component range from 0 to 3, with higher values indicating poorer sleep quality. The final sleep quality index is obtained by adding the component scores together. The total score ranges from a minimum score of 0 to a maximum of 21, and a higher total score indicates poorer sleep quality. As in Buysee’s work [33], this study interpreted a total score of 5 or lower as indicating normal sleep quality, while a score of 6 or higher indicated poor sleep quality.

### Statistical analysis

Using the independent-samples *t*-test, the participants’ serum vitamin D levels and mean scores for each PSQI component were measured. The chi-square test was used to analyze differences in the distribution of serum vitamin D deficiency and sleep quality. Simple logistic regression analysis was used to identify variables that showed a significant effect on sleep quality. Variables that were found to be significant were adjusted for in the multiple logistic regression analysis. SPSS version 21 (IBM Corp., Armonk, NY, USA) was used for the statistical analysis; the confidence level was set at 95% and significance level at *P* < 0.05.

## Results

### Subject characteristics

A total of 1472 participants were analyzed in this study. The mean serum vitamin D level in all participants was 13.70 ± 5.93 ng/mL. Of the participants, 813 (55.2%) were 30 years of age or older, while 659 (44.8%) were younger than 30. Males comprised 1178 (80.0%) of the participants, while 294 (20.0%) subjects were females. A total of 535 (36.3%) participants reported exercising ≥3 times a week, while 937 (63.7%) exercised <3 times per week. Current smokers included 428 (29.1%) of the participants, and 1044 (70.9%) participants were never smokers or former smokers. The high-risk drinking category contained 384 (26.1%) participants, while 1088 (73.9%) participants were in the normal drinking group. Of the participants, 524 (35.6%) had a body mass index ≥25 kg/m^2^, while 948 (64.4%) had a body mass index <25.0 kg/m^2^. There were 774 (52.6%) married participants and 698 (47.4%) unmarried participants. An education level of high school graduation or less was reported by 478 (32.5%) persons, while 994 (67.5%) reported a bachelor’s degree or higher. An employee tenure of less than 5 years was reported by 468(31.8%) participants, a tenure of 5–9 years was reported by 383 (26.0%), and over 10 years by 621 (42.2%). Higher than median occupational stress was reported by 321 subjects (21.8%), while 1152 (78.2%) reported occupational stress levels lower than the standard median. The participants were tested with the following seasonal distribution: 300 (20.4%) participants in spring, 456 (31.0%) in summer, 150 (10.2%) in autumn, and 566 (38.5%) in winter (Table 1).

**Table 1:** Subject characteristics

Variables		Number (%)		Vitamin D (ng/mL)	*P* value^a^
				Mean (SD)^b^	
Total		1472	(100)	13.70 5.93	
Age, years	≥30	813	(55.2)	14.56 6.14	0.009
	<30	659	(44.8)	13.25 5.63	
Sex	Male	1178	(80.0)	14.29 6.00	<0.001
	Female	294	(20.0)	11.31 4.95	
Regular exercise, times per week	≥3	535	(36.3)	14.37 6.09	0.001
	<3	937	(63.7)	13.31 5.80	
Smoking	Current	428	(29.1)	14.83 6.42	<0.001
	Never/former	1044	(70.9)	13.23 5.65	
Risky drinking^b^	Yes	384	(26.1)	14.51 5.77	0.002
	No	1088	(73.9)	13.41 6.28	
Body mass index, kg/m^2^	<25	948	(64.4)	13.69 6.03	0.995
	≥25	524	(35.6)	13.70 5.74	
Marital status	Married	774	(52.6)	14.31 6.13	<0.001
	Unmarried	698	(47.4)	13.01 5.62	
Education level	≥College	994	(67.5)	13.83 5.94	0.20
	≤Highs chool	478	(32.5)	13.41 5.89	
Job tenure, years	<5	468	(31.8)	12.98 5.59	0.001
	5–9	383	(26.0)	14.51 6.35	
	≥10	621	(42.2)	13.73 5.84	
Job stress	Low	1152	(78.2)	13.72 5.95	0.766
	High	321	(21.8)	13.61 5.86	
Test season^c^	Spring	300	(20.4)	12.53 5.09	0.001
	Summer	456	(31.0)	16.87 6.11	
	Autumn	150	(10.2)	14.52 5.86	
	Winter	566	(38.5)	11.54 5.93	

^a^Comparison made using the Student t-test

^b^Risky drinking: 2 or more times per week and 7 (females: 5) or more drinks per drinking session

^c^Spring, March–May; summer, June–August; autumn, September–November; winter, December–February

### Serum vitamin D levels of the subjects

A serum vitamin D deficiency (<10 ng/mL) was observed in 432 (29.3%) participants, while 1040 (70.7%) had serum vitamin D levels ≥10 ng/mL. Serum vitamin D deficiency showed a significant association with gender (*P* < .001), as it was found in 24.8% of males and 47.6% of females. Similarly, serum vitamin D deficiency was significantly less common in those who exercised ≥3 times per week (24.3%) than in those who exercised <3 times per week (32.2%) (*P* = .001). Serum vitamin D deficiency was significantly less common in current smokers (23.4%) than in former/never smokers (31.8%) (*P* = .001). Of high-risk drinkers, 22.9% exhibited serum vitamin D deficiency, which was significantly lower than the proportion in the normal drinking group (31.6%; *P* = .001). Serum vitamin D deficiency was found in 32.8% of unmarried participants and 26.2% of married subjects (*P* = .006). Serum vitamin D deficiency was found in 34.0% of participants tested in the spring, 12.3% of those tested in the summer, 22.0% of those tested in the autumn, and 42.6% of those tested in the winter; serum vitamin D deficiency was found to significantly more common in the winter group (*P* < .001). Age, body mass index, education level, employee tenure, and occupational stress did not show a significant difference in serum vitamin D deficiency distribution (Table 2).

**Table 2:** Serum vitamin D levels of the subjects

Variables		Vitamin D				*P* value^a^
		≥10 ng/mL		<10 ng/mL		
		*N*	%	*N*	%	
Total		1040	(70.7)	432	(29.3)	
Age, years	≥30	584	(71.8)	229	(28.2)	0.269
	<30	456	(69.2)	203	(30.8)	
Sex	Male	886	(75.2)	292	(24.8)	<0.001
	Female	154	(52.4)	140	(47.6)	
Regular exercise, times per week	≥3	405	(75.7)	130	(24.3)	0.001
	<3	635	(67.8)	302	(32.2)	
Smoking	Current	328	(76.6)	100	(23.4)	0.001
	Never/former	712	(68.2)	332	(31.8)	
Risky drinking^b^	Yes	296	(77.1)	88	(22.9)	0.001
	No	744	(68.4)	344	(31.6)	
Body mass index, kg/m^2^	<25	668	(70.5)	280	(29.5)	0.831
	≥25	372	(71.0)	152	(29.0)	
Marital status	Married	571	(73.8)	203	(26.2)	0.006
	Unmarried	469	(67.2)	229	(32.8)	
Education level	≥College	718	(72.2)	276	(27.8)	0.055
	≤High school	322	(67.4)	156	(32.6)	
Job tenure, years	<5	321	(68.6)	147	(31.4)	0.387
	5–9	277	(72.3)	106	(27.7)	
	≥10	442	(71.2)	179	(28.8)	
Job stress	Low	807	(70.1)	344	(29.9)	0.390
	High	233	(72.6)	88	(27.4)	
Test season^c^	Spring	198	(66.0)	102	(34.0)	<0.001
	Summer	400	(87.7)	56	(12.3)	
	Autumn	117	(78.0)	33	(22.0)	
	Winter	325	(57.4)	241	(42.6)	

^a^Comparison made using the chi-square test

^b^Risky drinking: 2 or more times per week and 7 (females: 5) or more drinks per drinking session

^c^Spring, March–May; summer, June–August; autumn, September–November; winter, December–February

*Abbreviations: SD* standard deviation

### Distribution of sleep quality according to general characteristics and serum vitamin D levels

Good sleep quality was reported by 1152 (78.3%) participants, while 319 (21.7%) reported poor sleep quality. Poor sleep quality was experienced by 19.8% of participants with adequate serum vitamin D levels and by 26.2% of participants with serum vitamin D deficiency (*P* = .007). Poor sleep quality was more common in those younger than 30 years of age (26.3%) than in those 30 years of age or older (18.0%) (*P* < .001). Poor sleep quality was significantly more common (*P* = .006) in females (27.6%) than in males (20.2%). Unmarried participants were more likely to have poor sleep quality (26.2%) than married participants (17.6%) (*P* < .001). Participants with a high school education or less reported poor sleep quality more frequently (27.2%) than those with a bachelor’s degree or higher (19.0%) (*P* < .001). Those who had worked for 5–9 years had the highest prevalence of poor sleep quality (24.5%), while those with a work tenure of 10 years or more had the lowest prevalence of poor sleep quality (18.5%) (*P* = .037). Those who reported high levels of occupational stress exhibited poor sleep quality more frequently (29.3%) than those with low levels of occupational stress (19.5%) (*P* < .001). Regular exercise, smoking habits, alcohol consumption habits, body mass index, and testing season were not associated with significant differences in sleep quality (Table 3).

**Table 3:** Distribution of sleep quality according to general characteristics and serum vitamin D levels

Variables		PSQI score^a^				*P* value^b^
		<6		≥6		
		N	%	N	%	
Total		1153	(78.3)	319	(21.7)	
Vitamin D,	≥10	834	(80.2)	206	(19.8)	0.007
ng/mL	<10	319	(73.8)	113	(26.2)	
Age, years	≥30	667	(82.0)	146	(18.0)	<0.001
	<30	486	(73.7)	173	(26.3)	
Sex	Male	940	(79.8)	238	(20.2)	0.006
	Female	213	(72.4)	81	(27.6)	
Regular exercise, times per week	≥3	422	(78.9)	113	(21.1)	0.699
	<3	731	(78.0)	206	(22.0)	
Smoking	Current	329	(76.9)	99	(23.1)	0.384
	Never/former	824	(78.9)	220	(21.1)	
Risky drinking^c^	Yes	287	(74.7)	97	(25.3)	0.052
	No	866	(79.6)	222	(20.4)	
Body mass index, kg/m^2^	<25	737	(77.7)	211	(22.3)	0.463
	≥25	416	(79.4)	108	(21.7)	
Marital status	Married	638	(82.4)	136	(17.6)	<0.001
	Unmarried	515	(73.8)	183	(26.2)	
Education level	≥College	805	(81.0)	189	(19.0)	<0.001
	≤High school	348	(72.8)	130	(27.2)	
Job tenure, years	<5	358	(76.5)	110	(23.5)	0.037
	5–9	289	(75.5)	94	(24.5)	
	≥10	506	(81.5)	115	(18.5)	
Job stress	Low	962	(80.5)	225	(19.5)	<0.001
	High	227	(70.7)	94	(29.3)	
Test season^d^	Spring	243	(81.0)	57	(19.0)	0.257
	Summer	355	(77.9)	101	(22.1)	
	Autumn	119	(79.3)	31	(20.7)	
	Winter	436	(77.0)	130	(23.0)	

^a^Pittsburgh Sleep Quality Index

^b^Comparison made using the chi-square test

^c^Risky drinking: 2 or more times per week and 7 (females: 5) or more drinks per drinking session

^d^Spring, March–May; summer, June–August; autumn, September–November; winter, December–February

### Simple and multiple logistic regression analysis of factors affecting sleep quality

Logistic regression analysis was used to identify variables with a effect on sleep quality. Simple logistic regression analysis showed an odds ratio (OR) of 1.43 (95% CI, 1.10–1.87) for having poor sleep quality in those with serum vitamin D deficiency. High risks of poor sleep quality were also found in other groups: those younger than 30 years of age (OR = 1.63; 95% CI, 1.27–2.09), females (OR = 1.50; 95% CI, 1.12–2.01), unmarried subjects (OR = 1.67; 95% CI, 1.30–2.14), those with a high school education or less (OR = 1.59; 95% CI, 1.23–2.06), and those with high occupational stress (OR = 1.55; 95% CI, 1.15–2.09). Employee tenure of 10 or more years was associated with a lower risk of poor sleep quality (OR = 0.74; 95% CI, 0.55–0.99) than those with <5 years of tenure. Variables in the simple logistic regression analysis were adjusted for in the multiple logistic regression analysis. The resulting OR was 1.36 (95% CI, 1.01–1.82) for poor sleep quality in the serum vitamin D deficiency group. Statistical significance was also found for being unmarried (OR = 1.47; 95% CI, 1.04–2.08), having a high school education or less (OR = 1.32; 95% CI, 1.03–1.77), and high occupational stress (OR = 1.85; 95% CI, 1.38–2.47). However, age, sex, and employee tenure were not found to be significant in the multiple logistic regression analysis (Table 4).

**Table 4:** Simple and multiple logistic regression analysis of factors affecting sleep quality

Variables		Unadjusted		Adjusted^a^	
		OR^b^	95% CI^c^	OR^b^	95% CI^c^
Vitamin D,	≥10	1.00		1.00	
ng/mL	<10	1.43	1.10–1.87	1.36	1.01–1.82
Age, years	≥30	1.00		1.00	
	<30	1.63	1.27–2.09	1.26	0.87–1.83
Sex	Male	1.00		1.00	
	Female	1.50	1.12–2.01	1.49	0.99–2.09
Regular exercise,	≥3	1.00		1.00	
times per week	<3	1.05	0.81–1.36	1.04	0.80–1.37
Smoking	Never/former	1.00		1.00	
	Curren	1.13	0.86–1.48	1.21	0.90–1.64
Risky drinking^d^	No	1.00		1.00	
	Yes	1.34	0.95–1.76	1.40	0.97–1.87
Body mass index,	<25	1.00		1.00	
kg/m^2^	≥25	0.91	0.70–1.18	1.01	0.76–1.33
Marital status	Married	1.00		1.00	
	Unmarried	1.67	1.30–2.14	1.47	1.04–2.08
Education level	≥College	1.00		1.00	
	≤High school	1.59	1.23–2.06	1.32	1.03–1.77
Job tenure, years	<5	1.00		1.00	
	5–9	1.06	0.77–1.45	1.23	0.85–1.77
	≥10	0.74	0.55–0.99	1.04	0.69–1.56
Job stress	Low	1.00		1.00	
	High	1.55	1.15–2.09	1.85	1.38–2.47
Test season^e^	Spring	1.00		1.00	
	Summer	1.21	0.84–1.74	1.14	0.76–1.70
	Autumn	1.11	0.68–1.81	1.14	0.69–1.90
	Winter	1.27	0.90–1.80	1.21	0.84–1.74

^a^Adjusted for vitamin D serum concentration, age, sex, regular exercise, smoking, risky drinking, body mass index, marital status, education level, job tenure, and job stress, test season

^b^Odds ratio

^c^Confidence interval

^d^Risky drinking: 2 or more times per week and 7 (females: 5) or more drinks per drinking session

^e^Spring, March–May; summer, June–August; autumn, September–November; winter, December–February

### Differences in the overall PSQI score and in each item of the PSQI according to serum vitamin D levels

The mean total PSQI score of all participants was 3.97 ± 2.26. Subjects in the serum vitamin D deficiency group showed a mean PSQI score of 4.25 ± 2.37, while those with serum vitamin D levels ≥10 ng/mL averaged 3.85 ± 2.15 (*P* = .002). The mean scores for each PSQI component were as follows: subjective sleep quality was poorer in those with vitamin D deficiency (1.05 ± 0.63, vs. 0.96 ± 0.61 in the non-deficient group) (*P* = .020), sleep latency was poorer in those with vitamin D deficiency (0.84 ± 0.85, vs. 0.64 ± 0.75 in the non-deficient group) (*P* < .001), and the sleep duration score was poorer in those with vitamin D deficiency (1.10 ± 0.65, vs. 1.03 ± 0.62 in the non-deficient group) (*P* = .041). Significant differences in the mean score were not found for the remaining categories of habitual sleep efficiency, sleep disturbances, use of sleep medication, and daytime dysfunction (Table 5).

**Table 5:** Differences in the overall PSQI score and the scores for each item according to serum vitamin D level

Variables	Mean (SD)	Vitamin D				*P* value^a^
		≥10 ng/mL		<10 ng/mL		
		Mean (SD)		Mean (SD)		
PSQI total score	3.97 2.26	3.85	2.15	4.25	2.37	0.002
Subjective sleep quality	0.99 0.61	0.96	0.61	1.05	0.63	0.020
Sleep latency	0.70 0.79	0.64	0.75	0.84	0.85	<0.001
Sleep duration	1.05 0.63	1.03	0.62	1.10	0.65	0.041
Habitual sleep efficiency	0.03 0.23	0.03	0.24	0.04	0.22	0.587
Sleep disturbances	0.63 0.50	0.62	0.49	0.64	0.51	0.613
Use of sleep medication	0.01 0.13	0.01	0.12	0.01	0.14	0.396
Daytime dysfunction	0.77 0.73	0.76	0.73	0.79	0.74	0.526

^a^Comparison made using the Student t-test

*Abbreviations: PSQI* Pittsburgh Sleep Quality Index, *SD* standard deviation

## Discussion

In this study, we investigated the prevalence of serum vitamin D deficiency and poor sleep conditions in fixed day indoor field workers in the manufacturing industry in Korea in order to obtain information regarding their general sleeping conditions and the relationship of vitamin D with sleep quality.

We found that the mean serum vitamin D level in participants was generally low (13.70 ng/mL), with lower levels found in females (11.31 ng/mL) than in males (14.29 ng/mL). This is lower than the mean serum vitamin D level of 17.38 ng/mL that was found in an analysis of KNHANES data (KNHANES 2010–2011) [3]. These results are also lower than those of another study investigating the association between vitamin D and occupational factors in Korean workers [34], in which mean levels of 17.80 ng/mL in males and 15.60 ng/mL in females were reported. A possible explanation for the low vitamin D levels in the current study is that the sample of this study was daytime indoor workers who were more likely to have low exposure to sunlight than the general Korean working population. Unlike previous studies that found lower vitamin D levels in younger groups due to more time spent on indoor activities and the frequent use of sunscreen in that population [34, 35], this study did not find a significant difference in vitamin D levels according to age. This may have been due to the narrow age range of the study subjects (19–39 years). Other variables that were associated with significant differences in serum vitamin D levels were regular exercise, smoking habits, alcohol consumption habits, marital status, and testing season. Exercise is known to help the synthesis of vitamin D in the skin through ultraviolet radiation exposure [36] but this study did not investigate whether exercise incorporated outdoor activities. However, it can be assumed that the group that exercised 3 or more times a week had more opportunities for sunlight exposure, based on their higher serum vitamin D levels. Furthermore, smoking and alcohol consumption are known to affect parathyroid hormone, which controls the absorption of calcium [37, 38], ultimately causing vitamin D deficiency. In this study, however, the smoking and drinking groups showed higher levels of serum vitamin D. This outcome most likely reflects the disparity in the sex of the subjects, with the majority being male [39]. Males were more common in the groups of current smokers and subjects who engaged in high-risk drinking. Serum vitamin D levels were found to be highest during the summer months (June–August), with a mean level of 16.87 ng/mL, and lowest in the winter (December–February), with a mean level of 11.54 ng/mL. This result is consistent with previous studies that reported lower serum vitamin D levels in the winter, since vitamin D synthesis in the skin depends on the amount of sunlight [5].

The mean PSQI score of all subjects in this study was 3.97 ± 2.26, and poor sleep quality was found in 21.7% of respondents. A previous study on 263 paramedics working with Korea’s 119 rescue service that used PSQI as a tool to measure sleep quality [40] reported that the mean PSQI score was 7.73, with 68.4% of respondents reporting poor sleep quality. Another study that similarly investigated 2818 electronics manufacturing industry workers [41] found a mean PSQI score of 5.95, with 50.1% of subjects experiencing poor sleep quality. The mean PSQI score and proportion of respondents with poor sleep quality in the current study were relatively low compared to previous studies. A possible explanation for such results is that in the design of this study, nighttime shifts and symptoms of depression were excluded as exogenous variables [41, 42] and did not affect the results.

Age, sex, marital status, education level, employee tenure, and occupational stress were adjusted for in the logistic regression analysis conducted to assess the correlation between vitamin D and sleep, and a significant association was found between serum vitamin D deficiency and poor sleep quality (OR = 1.33; 95% CI, 1.01–1.76). This result is dissimilar to those of previous studies that did not show significant correlations, such as a cohort study on the correlation between vitamin D deficiency and daytime sleepiness in United States sleep clinic patients [9] and a study of serum vitamin D levels and sleep conditions in pregnant women in Turkey [43]. It is difficult to generalize and compare these study results because both studies focused on specific considerations, such as a specific sleep disorder or specific research subjects such as pregnant women, and also were small-scale studies conducted on fewer than 100 subjects. However, a study on men over the age of 65 [44] was published in the *Journal of Sleep*. That study measured objective sleep duration and sleep effectiveness using wrist actigraphy and found that lower serum vitamin D levels were associated with shorter sleep duration and poorer sleep effectiveness, consistent with the findings of the present study.

The precise mechanism of vitamin D and its effect on sleep is still unclear, except that vitamin D is likely to play a major role in the brainstem, which controls sleep [19]. This hypothesis is supported by evidence of vitamin D receptors in parts of the brainstem such as the anterior and posterior hypothalamus, substantia nigra, midbrain central grey matter, raphe nuclei, and in the nucleus reticularis pontisoralis and caudalis. These regions are known to use pacemaker cells to execute important roles in the first stages of sleep and in maintaining sleep. Specifically, the hypothalamus and nucleus reticularis pontis suppress muscles that affect sleep, such as the bulbar and somatic musculature. Other effects of vitamin D on sleep were reported in Stumpf and Jennes’ 1984 study [45]. In that study, vitamin D was found to be a broad-range steroid hormone. Like all steroid hormones, it is involved with the endocrine and autonomic nervous systems, and as such is closely associated with brainstem regions such as the medulla oblongata, pontine nuclei, and midbrain nuclei. The role of vitamin D in the endocrine and autonomic nervous systems could lead to wide-ranging effects on the cardiovascular system, digestive system, immune system, and sleep-wake cycle.

Other variables that showed significant associations with poor sleep quality in this study were marital status (OR = 1.51; 95% CI, 1.07–2.12), education level (OR = 1.38; 95% CI, 1.04–1.82), and occupational stress (OR = 1.84; 95% CI, 1.38–2.45). The results showing that marriage was associated with improved sleep quality were consistent with Soltani et al.’s study finding that stable marital life improved sleep quality in women [46].

This study found that those with a high school education or less had worse sleep quality, similarly to previous research that found that discrimination and stress within the job environment due to lower education levels could cause sleep disorders [47]. The current study found occupational stress to be the most significant variable associated with sleep quality, and a previous study conducted on nurses also found that 97% of those who experienced moderate or higher occupational stress levels had poor sleep quality [48]. The current study categorized occupational stress groups using the KOSS-SF total score median reference value. Further investigation into sub-factors that could influence sleep quality, such as the scope of job competency, decision-making authority, role conflict, and employee-superior conflict should be incorporated into future studies.

Serum vitamin D levels were associated with significant differences in the total PSQI score, subjective sleep quality, sleep latency, and sleep duration. A study of United States veterans and the effects of vitamin D supplementation on pain and sleep [49] similarly found that those with vitamin D deficiency had higher total PSQI scores than the normal group, as well as longer sleep latency and shorter sleep duration. Furthermore, that study found vitamin D supplementation to significantly improve sleep quality in terms of the total PSQI score, sleep latency, and sleep duration in both the vitamin D deficiency group and the group with normal vitamin D levels. Research in Korea, however, has mainly been focused on the relationship between vitamin D and sleep duration [23, 24], making it difficult to identify the general effects of vitamin D on sleep in Koreans. Given this background, this study collected participants’ sleep data using the PSQI in order to identify the various effects vitamin D has on sleep in addition to sleep duration.

This study identified a correlation between serum vitamin D and sleep quality, but encountered limitations in explaining the exact causal relationship due to the cross-sectional design of the study. Furthermore, although structured surveys were used for data collection, they were self-report surveys that may have been biased, potentially influencing the research results. Additionally, the research subjects were identified as indoor workers in an electronics manufacturing workplace, but each worker’s outdoor physical activities and their dietary habits, such as whether they took vitamin D supplements, could not be investigated. Despite these limitations, as a single-institution study, this study was conducted with the same examiners, facilities, and methods. Moreover, this study attempted to minimize the influence of outdoor activities on the results by limiting the subjects to indoor workers. The final limitation of this study is that the subjects were drawn from the working population, which makes it difficult to generalize the study results to the general population. These limitations will need to be addressed in future research.

## Conclusions

Previous studies have found serum vitamin D levels to be critically low in Koreans compared to other countries [3–6], and have found a continuous increase in sleep-related disorders [19]. However, research into the relationship between serum vitamin D levels and sleep quality in Korean subjects has not been conducted. This study filled that gap by researching a large number of Korean workers and their serum vitamin D levels, and characterizing the associations of serum vitamin D levels with sleep. But, the statistical significance of relatively low serum vitamin D levels and sleep quality may be a limitation. Many factors influence sleep, and serum vitamin D deficiency cannot be the only factor that explains sleep quality. Even so, this study proved that serum vitamin D deficiency was associated with sleep quality, in addition to other factors. Based on these results, workplaces can manage sleep disorders through regular serum vitamin D deficiency examinations combined with appropriate care in order to more effectively prevent sleep disorders. Furthermore, it is necessary to conduct research on the effect of active serum vitamin D treatment on improving sleep symptoms in individuals with serum vitamin D deficiency and poor sleep quality.

## Abbreviations

CI: Confidence interval
KNHANES: Korean National Health and Nutrition Examination Survey
OR: Odds ratio
PSQI: Pittsburgh Sleep Quality Index
SD: Standard deviation
SPSS: Statistical Package for the Social Sciences

## References

[1] Norman AW, Bouillon R (2010). Vitamin D nutritional policy needs a vision for the future. Exp Biol Med.

[2] Holick MF, Chen TC (2008). Vitamin D deficiency: a worldwide problem with health consequences. Am J ClinNutr.

[3] Jung IK (2013). Prevalence of vitamin Deficiency in Korea: Results from KNHANES 2010 to 2011. J Nutr Health.

[4] Ganji V, Zhang X, Tangpricha V (2012). Serum 25-hydroxyvitamin D concentrations and prevalence estimates of hypovitaminosis D in the U.S. population based on assay-adjusted data. J Nutr.

[5] Langlois K, Greene-Finestone L, Little J, Hidiroglou N, Whiting S (2010). Vitamin D status of Canadians as measured in the 2007 to 2009 Canadian Health Measures Survey. Health Rep.

[6] Ono Y, Suzuki A, Kotake M, Zhang X, Nishiwaki-Yasuda K, Ishiwata Y, Itoh M (2005). Seasonal changes of serum 25-hydroxyvitamin D and intact parathyroid hormone levels in a normal Japanese population. J Bone Miner Metab.

[7] Nair R, Maseeh A (2012). Vitamin D: The “sunshine” vitamin. J Pharmacol Pharmacother.

[8] Holick MF (2007). Vitamin D deficiency. N Engl J Med.

[9] McCarty DE, Reddy A, Keigley Q, Kim PY, Marino AA (2012). Vitamin D, race, and excessive daytime sleepiness. J Clin Sleep Med.

[10] Moan J, Porojnicu AC, Dahlback A, Setlow RB (2008). Addressing the health benefits and risks, involving vitamin D or skin cancer, of increased sun exposure. Proc Natl Acad Sci U S A.

[11] Webb AR (2006). Who, what, where and when—influences on cutaneous vitamin D synthesis. Prog Biophys Mol Biol.

[12] Lips P (2001). Vitamin D deficiency and secondary hyperparathyroidism in the elderly: consequences for bone loss and fractures and therapeutic implications. Endocr Rev.

[13] Wengreen HJ, Munger RG, West NA, Cutler DR, Corcoran CD, Zhang J, Sassano NE (2004). Dietary protein intake and risk of osteoporotic hip fracture in elderly residents of Utah. J Bone Miner Res.

[14] Zhao G, Ford ES, Li C, Croft JB (2012). Serum 25-hydroxyvitamin D levels and all-cause and cardiovascular disease mortality among US adults with hypertension: the NHANES linked mortality study. J Hypertens.

[15] Wolden-Kirk H, Overbergh L, Christesen HT, Brusqaard K, Mathieu C (2011). Vitamin D and Diabetes: its importance for beta cell and immune function. Mol Cell Endocrinol.

[16] Renzaho AM, Halliday JA, Nowson C (2011). Vitamin D, obesity, and obesity-related chronic disease among ethnic minorities: a systematic review. Nutrition.

[17] Chen TC, Holick MF (2003). Vitamin D and prostate cancer prevention and treatment. Trends Endocrinol Metab.

[18] Kamen D (2010). L, Vin T. Vitamin D and molecular actions on the immune system: modulation of innate and autoimmunity. J Mol Med.

[19] Gominak SC, Stumpf WE (2012). The world epidemic of sleep disorders is linked to vitamin D deficiency. Med Hypotheses.

[20] Skaer TL, Sclar DA (2010). Economic implications of sleep disorders. Pharmaco Economics.

[21] Lee J, Lee JS, Park SH, Shin SA, Kim K. Cohort Profile: The National Health Insurance Service–National Sample Cohort (NHIS-NSC), South Korea. Int J Epidemiol. 2016;dyv319. 10.1093/ije/dyv319 26822938

[22] Lee HJ, Lee HD, Hwang HS, Park HK, Kim SE (2014). Associations between Serum Vitamin D Level and Mean Hours of Sleep: 2011 Korea National Health and Nutrition Examination. Survey Korean J Fam Pract.

[23] Kim JH, Chang JH, Kim DY, Kang JW (2014). Association Between Self-Reported Sleep Duration and Serum Vitamin D Level in Elderly Korean Adults. J Am Geriatr Soc.

[24] Weisell RC (2002). Body mass index as an indicator of obesity. Asia Pac J Clin Nutr.

[25] Korea National Health and Nutrition Examination Survey (KNHANES VI-1). https://knhanes.cdc.go.kr/knhanes/index.do. Accessed 28 Jun 2016.

[26] Chang SJ, Koh SB, Kang D, Kim SA, Kang MG, Lee CG, Chung JJ, Cho JJ, Son M, Chae CH, Kim JW, Kim JI, Kim HS, Roh SC, Park JB, Woo JM, Kim SY, Kim JY, Ha M, Park J, Rhee KY, Kim HR, Kong JO, Kim IA, Kim JS, Park JH, Huyun SJ, Son DK (2005). Developing an occupational stress scale for Korean employees. Korean J Occup Environ Med.

[27] Norman AW (2008). From vitamin D to hormone D: fundamentals of the vitamin D endocrine system essential for good health. Am J Clin Nutr.

[28] Gómez-Alonso C, Naves-Díaz ML, Fernández-Martín JL, Díaz-López JB, Fernández-Coto MT, Cannata-Andía JB (2003). Vitamin D status and secondary hyperparathyroidism: The importance of 25-hydroxyvitamin D cut-off levels. Kidney Int Sippl.

[29] Karatekin G, Kaya A, Salihoğlu Ö, Balci H, Nuhoğlu A (2009). Association of subclinical vitamin D deficiency in newborns with acute lower respiratory infection and their mothers. Eur J ClinNutr.

[30] McKenna M, Freaney R (1998). Secondary Hyperparathyroidism in the Elderly: Means to Defining Hypovitaminosis D. Osteoporos Int.

[31] Hamilton B, Grantham J, Racinais S, Chalabi H (2010). Vitamin D deficiency is endemic in Middle Eastern sportsmen. Public Health Nutr.

[32] Kim GD. A study on quality of sleep and sleep disturbing factors among community dwelling elderly. Korean J Soc Welfare Aged. 2000;7(1):173–192. Korean.

[33] Buysse DJ, Reynolds CF, Monk TH, Berman SR, Kupfer DJ (1989). The Pittsburgh sleep quality index: a new instrument for psychiatric practice and research. Psychiatry Res.

[34] Jeong H, Hong S, Heo Y, Chun H, Kim D, Park J, Kang MY (2014). Vitamin D status and associated occupational factors in Korean wage workers: data from the 5th Korea national health and nutrition examination survey (KNHANES 2010–2012). Ann Occup Environ Med.

[35] Kulie T, Groff A, Redmer J, Hounshell J, Schrager S (2009). Vitamin D: an evidence-based review. J Am Board Fam Med.

[36] Sadat-Ali M, Al Elq A, Al-Farhan M, Sadat NA (2013). Fortification with vitamin D: comparative study in the Saudi Arabian and US markets. J Fam Community Med.

[37] Cutillas-Marco E, Fuertes-Prosper A, Grant WB, Morales-Suárez-Varela M (2012). Vitamin D deficiency in South Europe: effect of smoking and aging. Photodermatol Photoimmunol Photomed.

[38] Naude CE, Carey PD, Laubscher R, Fein G, Senekal M (2012). Vitamin D and calcium status in South African adolescents with alcohol use disorders. Nutrients.

[39] Dam T, von Mühlen D, Barrett-Connor E (2009). Sex-specific association of serum vitamin D levels with physical function in older adults. Osteoporos Int.

[40] Yun SW, Oh K, Yun H, Park J (2013). Relationship between job stress and quality of sleep among 119 recue workers. J Korea Acad Ind Cooperation Soc.

[41] Kim JY, Chae CH, Kim YO, Son JS, Kim JH, Kim CW, Kwon SI (2015). The relationship between quality of sleep and night shift rotation interval. Ann Occup Environ Med.

[42] Yoshitaka K, Takashi O, Makoto U, Shinji T, Kazuo K, Eise Y, Takeo M, Satoru H, Kenshu S, Toshiharu F (2006). The Relationship Between Depression and Sleep Disturbances: A Japanese Nationwide General Population Survey. J Clin Psychiatry.

[43] Gunduz S, Kosger H, Aldemir S, Akcal B, Tevrizci H, Hizli D, Celik HT (2016). Sleep deprivation in the last trimester of pregnancy and inadequate vitamin D: Is there a relationship?. J Chin Med Assoc.

[44] Jennifer M, Katie L, Esther K, Stephanie L, Elizabeth BC, Nancy E, Misti P, Sonia AI, Eric O, Eva S (2015). Vitamin D and Actigraphic Sleep Outcomes in Older Community-Dwelling Men: The MrOS Sleep Study. Sleep.

[45] Stumpf WE, Jennes L (1984). The A-B-C (Allocortex-Brainstem-Core) circuitry of endocrine-autonomic integration and regulation: aproposed hypothesis on the anatomical-functional relationshipsbetween estradiol sites of action and peptidergic-aminergicneuronal systems. Peptides.

[46] Soltani M, Haytabakhsh MR, Najman JM, Williams GM, O'Callaghan MJ, Bor W, Dingle K, Clavarino A (2012). Sleepless nights: the effect ofsocioeconomic status, physical activity, andlifestyle factors on sleep quality in a largecohort of Australian women. Arch Womens Ment Health.

[47] Agargun MY, Besiroglu L (2005). Sleep and suicidality: do sleep disturbances predict suicide risk. Sleep.

[48] Najafi GT, Moradi F, Rafii F, Haghani H (2014). Relationship between Job Stress, Sleep Quality and Fatigue in Nurses. Iran J Nurs.

[49] Huang W, Shah S, Long Q, Crankshaw AK, Tangpricha V (2013). Improvement of pain, sleep, and quality of life in chronic pain patients with vitamin D supplementation. Clin J Pain.

